# Longitudinal Patterns in Antithrombotic Therapy in Patients with Atrial Fibrillation after Percutaneous Coronary Intervention in the Non-Vitamin K Oral Anticoagulant Era: A Nationwide Population-Based Study

**DOI:** 10.3390/jcm10071505

**Published:** 2021-04-04

**Authors:** Jiesuck Park, Jin-Hyung Jung, Eue-Keun Choi, Seung-Woo Lee, Soonil Kwon, So-Ryoung Lee, Jeehoon Kang, Kyung-Do Han, Kyung-Woo Park, Seil Oh, Gregory Y. H. Lip

**Affiliations:** 1 Department of Internal Medicine, Seoul National University Hospital, Seoul 03080, Korea; cardio.jspark@gmail.com (J.P.); david.soonil.kwon@gmail.com (S.K.); minerva1368@gmail.com (S.-R.L.); medikang@gmail.com (J.K.); kwparkmd@snu.ac.kr (K.-W.P.); seil@snu.ac.kr (S.O.); 2 Department of Internal Medicine, College of Medicine, Seoul National University, Seoul 03080, Korea; Gregory.Lip@liverpool.ac.uk; 3 Department of Medical Statistics, College of Medicine, The Catholic University of Korea, Seoul 06591, Korea; jungjin115@naver.com (J.-H.J.); hkd917@naver.com (K.-D.H.); 4 Department of Statistics and Actuarial Science, Soongsil University, Seoul 06978, Korea; ghdk32@naver.com; 5 Liverpool Centre for Cardiovascular Science, University of Liverpool, Liverpool Chest & Heart Hospital, Liverpool L14 3PE, UK; 6 Department of Clinical Medicine, Aalborg University, 9000 Aalborg, Denmark

**Keywords:** atrial fibrillation, percutaneous coronary intervention, anticoagulation, antiplatelets, non-vitamin K oral anticoagulant

## Abstract

We investigated whether longitudinal patterns in antithrombotic therapy have changed after the introduction of non-vitamin K oral anticoagulants (NOACs) in patients with atrial fibrillation (AF) who underwent percutaneous coronary intervention (PCI). Using a claims database of the Korean AF population who underwent PCI between 2012 and 2016 (*n* = 18,691), we analyzed prescription records of oral anticoagulants (OACs) and antiplatelets at 3-month intervals over 2 years after PCI. The study population was stratified (pre-NOAC, transition, and NOAC era) using time-periods of NOAC introduction in Korea and an expansion of reimbursement for NOAC in AF as indicators. The overall rates of OAC were low at baseline (24.9%, 26.9%, and 35.2% in pre-NOAC, transition, and NOAC era, respectively), contrary to high rates of dual antiplatelet therapy (DAPT) (73.3%, 71.4%, and 63.6%). However, OAC prescription rates were increased at 1-year (18.5%, 22.5%, and 31.6%), and 2-year follow-up (17.8%, 24.2%, and 31.8%) from pre-NOAC to NOAC era. In NOAC era, 63.5% of baseline OAC prescriptions comprised NOAC, of which 96.4% included triple therapy with DAPT. Over 2 years, we observed increasing rates of double therapy with a single antiplatelet (18.3% and 20.0% at 1- and 2-year follow-up) and OAC monotherapy (2.7% and 8.9% at 1- and 2-year follow-up).

## 1. Introduction

Combination therapy with anticoagulants and antiplatelets has been a major consideration in treatment decisions for patients with atrial fibrillation (AF) who undergo percutaneous coronary intervention (PCI) [1,2]. Guidelines recommend the initial administration of oral anticoagulants (OACs) for stroke prevention in addition to dual antiplatelet therapy (DAPT) after PCI (triple therapy (OACs plus DAPT)) [1,2]. However, the augmented risk of major bleeding associated with combination therapy may counterbalance the treatment benefits [3], and clinicians might be hesitant to administer guideline-based antithrombotic therapy after PCI [4,5]. Therefore, careful evaluation of treatment compliance is important in real-world clinical practice to guide clinicians for optimal management. A nationwide study can provide valuable longitudinal data regarding antithrombotic therapy in patients with AF who undergo PCI, because this information will aid in evaluation of long-term compliance [6]. Since non-vitamin K oral anticoagulants (NOACs) have been the major choice for stroke prevention in AF, a growing body of evidence has reported the outcome benefit of a combination regimen based on NOACs for patients with AF undergoing PCI [7,8,9,10]. Therefore, for those eligible for NOACs, guidelines recommend NOACs in preference to vitamin K antagonists (VKAs) in a combination regimen with antiplatelet therapy after PCI [1,2]. The current study was purposed to investigate whether the updated guidelines have changed the long-term treatment patterns in antithrombotic therapy among the AF population after PCI.

## 2. Materials and Methods

### 2.1. Study Population

Clinical data of the study population were obtained from the claims database of the Korean Health Insurance Review and Assessment Service (HIRA). The HIRA includes comprehensive details regarding complete medical healthcare utilization and cost data from primary care health services, pharmacies, and hospitals that cater to the entire Korean population [6]. The original database can be accessed via request through the Healthcare Bigdata Hub (https://opendata.hira.or.kr (accessed on 1 June 2020)) of the HIRA. From the database, we included patients with AF who underwent PCI between 2012 and 2016. Patients with AF were defined based on the International Classification of Diseases, Tenth Revision, Clinical Modification (ICD-10-CM) codes I48.0–48.4 and I48.9, excluding those with mitral stenosis (I50, I52, and I59) or prosthetic heart valves (Z952–Z954). Among patients with AF, we identified those who underwent PCI after the diagnosis of AF using the procedure codes for coronary intervention: M6551–6552, M6561–6564, and M6571–6572. We excluded patients who died before discharge and those for whom follow-up medication records were unavailable. Finally, we analyzed the data of 18,691 patients. NOACs were introduced in Korea in 2013 and have been widely used only since July 2015 after the expansion of the reimbursement criteria was adopted in Korea [4]. Therefore, we categorized the study population into the following cohorts based on the period of study inclusion: the pre-NOAC era (January 2012–June 2013), the transition era (July 2013–June 2015), and the NOAC era (July 2015–December 2016). The study was approved by the Seoul National University Hospital Institutional Review Board (E-1911-052-1078). Informed consent was waived by the review board as each patient is de-identified and encrypted in the HIRA database to protect patient privacy.

### 2.2. Antithrombotic Therapies

We reviewed prescription records of antithrombotic therapy, including OACs (VKAs or NOACs) and antiplatelet agents (aspirin and P2Y_12_ inhibitors comprising clopidogrel, prasugrel, and ticagrelor) for all patients in each cohort at a 3-month interval from the index PCI over a 2-year follow-up period. Based on prescription records, we categorized patients into the following groups: (1) triple therapy (VKAs or NOACs plus DAPT), (2) double therapy (VKAs or NOACs plus single antiplatelet therapy (SAPT)), (3) OACs monotherapy (VKAs or NOACs only), (4) DAPT, and (5) SAPT. Patients who died before 2 years or those who underwent repeat PCI before 2 years were censored at the event date.

### 2.3. Clinical Risk Factors

Detailed definitions of comorbidities and risk scores are summarized in Table S1. Hypertension and diabetes mellitus were defined based on both diagnostic codes and prescription claims for at least a single antihypertensive or antidiabetic drug, respectively. Congestive heart failure, stroke, systemic thromboembolism, myocardial infarction (MI), gastrointestinal (GI) bleeding, intracranial hemorrhage, and chronic liver or renal disease were defined based on ICD-10-CM codes. CHA_2_DS_2_-VASc scores were calculated for each patient to assess individual stroke risk. Data for the labile international normalized ratio and alcohol use are unavailable in the HIRA database. Therefore, we defined modified HAS-BLED scores after excluding these variables to assess the individual bleeding risk.

### 2.4. Statistical Analysis

An inter-cohort difference of baseline characteristics was assessed using one-way analysis of variance and Kruskal–Wallis test for continuous variables and chi-square tests for categorical variables, with multiple testing adjustments for pairwise comparison. We performed an inter-cohort comparison of the prescription rates of overall OACs and NOACs at 3-month intervals using the chi-square test. Therefore, we could analyze the 2-year patterns in antithrombotic therapy within each cohort and record any significant changes in these longitudinal treatment patterns from the pre-NOAC to the NOAC era.

In the previous nationwide study performed in the VKAs era [5], we observed that female sex, prior MI, and baseline DAPT use were independently associated with no OACs use 1 year after PCI. We also observed that most patients who received OACs were administered combination therapy that included antiplatelet agents instead of OAC monotherapy 1 year post-PCI. Therefore, we performed multivariable logistic regression analysis to identify the clinical factors associated with OAC use 1 year after PCI in the NOAC era. Additionally, we investigated the positive predictors of the preference for OAC monotherapy over combination regimens or antiplatelet-only therapy 1 year after PCI. We included all variables that showed statistical significance in univariate analysis and relevant factors associated with antithrombotic therapy in AF as covariates in the regression model. Collinearity was evaluated between the covariates, confirming no significant correlations. Individual model fitness was assessed using Hosmer–Lemeshow goodness-of-fit test.

All statistical analyses were performed using SAS software, version 9.3 (SAS Institute, Cary, NC, USA), and a two-tailed *p*-value < 0.05 was considered statistically significant.

## 3. Results

### 3.1. Baseline Characteristics

A total of 5044, 7209, and 6438 patients were included in the pre-NOAC, transition, and NOAC eras, respectively. Baseline characteristics of the three cohorts are summarized in Table 1. Patients in the pre-NOAC era were younger and had a lower comorbidities burden than those in the other cohorts. The CHA_2_DS_2_-VASc score (median (interquartile range)) was lowest for patients in the pre-NOAC era (3 (2–5)) and highest for those in the NOAC era (4 (2–5)). Baseline OACs prescription rates were 24.9%, 26.9%, and 35.2% in pre-NOAC, transition, and NOAC eras, respectively. The rate of NOACs was 0.4% in the pre-NOAC era, which significantly increased to 22.3% in the NOAC era. Triple therapy was the major type of combination regimen at baseline (24.3%, 26.2%, and 34.2%). In the NOAC era, NOAC were preferred over VKA as combination regimens in triple therapy (21.5% vs. 12.7%) and double therapy (0.7% vs. 0.1%). Most of the patients received DAPT without OAC at baseline (73.3%, 71.4%, and 63.6%) in all three cohorts. High DAPT rates at baseline were prominent among patients with previous MI (75.4%, 75.9%, and 66.6%) and those with peripheral artery disease (PAD) (74.9%, 73.6%, and 65.0%).

### 3.2. Two-Year Prescription Patterns in Antithrombotic Therapy after Percutaneous Coronary Intervention

**Figure 1:**
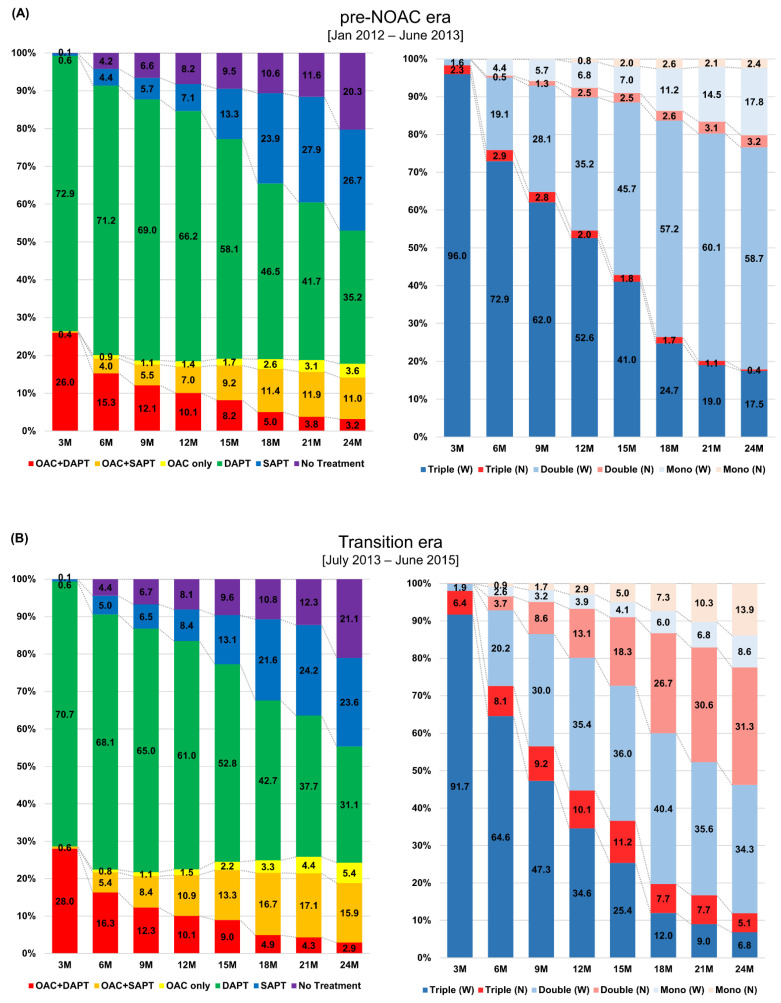

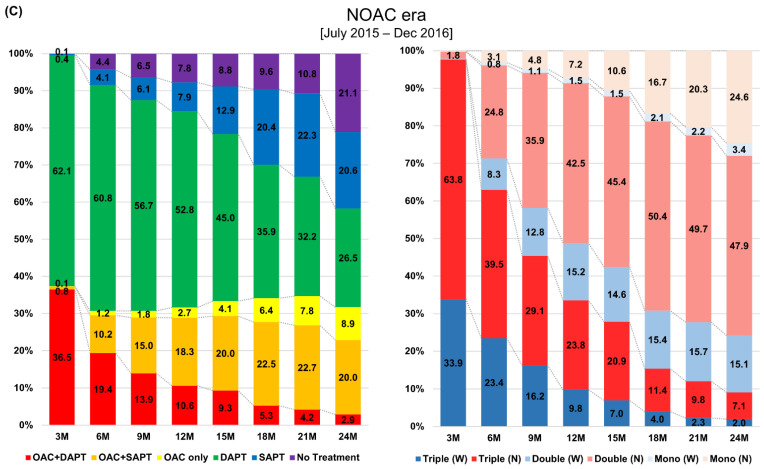
Two-year patterns in antithrombotic therapy after percutaneous coronary intervention in patients with atrial fibrillation. The figure represents 2-year patterns in antithrombotic therapy after PCI in pre-NOAC era (**A**), transition era (**B**), and NOAC era (**C**). In all three cohorts, prescription rates of OACs were substantially low over the 2-year period, whereas DAPT was the major type of antithrombotic therapy after PCI. (left panel of **A**–**C**). VKAs were the dominant type of OACs in the pre-NOAC and transition era. However, NOACs were preferred to VKAs in all OACs regimens in the NOAC era (right panel of **A**–**C**). DAPT: dual antiplatelet therapy; OAC: oral anticoagulant; N: non-vitamin K oral anticoagulant; PCI: percutaneous coronary intervention; SAPT: single antiplatelet therapy; W: warfarin.

In all three cohorts, the overall rates of OACs were substantially low over two years (18.5%, 22.5%, and 31.6% at 1-year period; 17.8%, 24.2%, and 31.8% at 2-year period in pre-NOAC, transition, and NOAC eras, respectively) (Figure 1). At the 1-year period, 73.3%, 69.4%, and 60.7% of patients in each cohort received antiplatelet therapy without OACs, most of which was DAPT (66.2%, 61.0%, and 52.8%). Similar rates were found at the 2-year period at which time 61.9%, 54.7%, and 47.1% of patients in each cohort had antiplatelet therapy without OACs (35.2%, 31.1%, and 26.5% were DAPT). The percentage of patients who did not receive any antithrombotic treatment (the no-treatment group) increased over 2 years; these percentages were 20.3%, 21.1%, and 21.1% at 2-year follow-up in each cohort.

**Figure 2:**
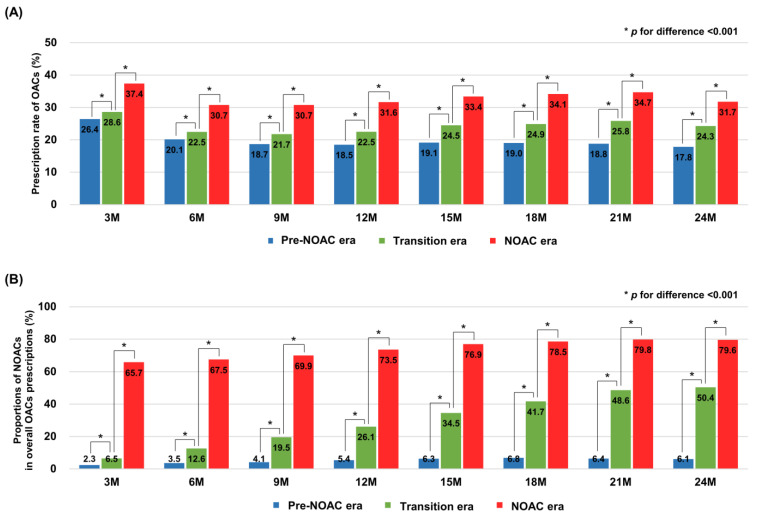
Comparison of prescription rates of overall oral anticoagulants and non-vitamin K oral anticoagulants between the study cohorts. The figure shows the prescription rates of OACs (**A**) and the proportions of NOACs in overall OACs prescriptions (**B**) compared between the three cohorts in every 3-month time interval. In each period, the prescription rates of OACs and the proportions of NOACs were significantly increased from pre-NOAC to NOAC era. OACs: oral anticoagulants; NOACs: non-vitamin K oral anticoagulants.

In terms of OACs regimens, triple therapy showed the highest proportions at 3-months after PCI in all three cohorts (26.0%, 28.0%, and 36.5%), which decreased over two years (10.1%, 10.1%, and 10.6% at 1-year period; 3.2%, 2.9%, and 2.9% at 2-year period). In contrast, the rates were increased over two years for double therapy (7.0%, 10.9%, and 18.3% at 1-year period; 11.0%, 15.9%, and 20.0% at 2-year period), and OAC monotherapy (1.4%, 1.5%, and 2.7% at 1-year period; 3.6%, 5.4%, and 8.9% at 2-year period). In the pre-NOAC era, VKAs were the major type of OACs in all regimens. At the 1-year period, 52.6% of OACs prescriptions were VKA-based triple therapy (vs. 2.0% of NOACs-based triple therapy), and 35.2% were VKA-based double therapy (vs. 2.5% of NOAC-based double therapy). VKA monotherapy accounted for 6.8% of OACs prescriptions at the 1-year period (vs. 0.2% of NOAC monotherapy). From the pre-NOAC to the NOAC era, the major type of OACs had been replaced from VKAs to NOACs. In the NOAC era, 42.5% of OACs prescriptions at the 1-year period were NOAC-based double therapy (vs. 15.2% of VKA-based double therapy), which increased to 47.9% (vs. 15.1%) at the 2-year period. The NOAC monotherapy rate was 7.2% (vs. 1.5% of VKA monotherapy) at the 1-year period, which increased to 24.6% (vs. 3.4%) at the 2-year period. When we compared the OACs prescription rates between the three cohorts at each 3-month time interval, the rates significantly increased from the pre-NOAC to the NOAC era (Figure 2). The proportions of NOACs in overall OACs prescriptions were also significantly increased from pre-NOAC to NOAC era. Table S2 summarizes the absolute numbers of patients with specific types of antithrombotic therapy in each cohort. Sensitivity analysis excluding all censored cases from the study population showed consistent patterns with those found in the main results (Figure S1).

### 3.3. Clinical Factors Associated with Oral Anticoagulants Use 1 Year after Percutaneous Coronary Intervention

**Figure 3:**
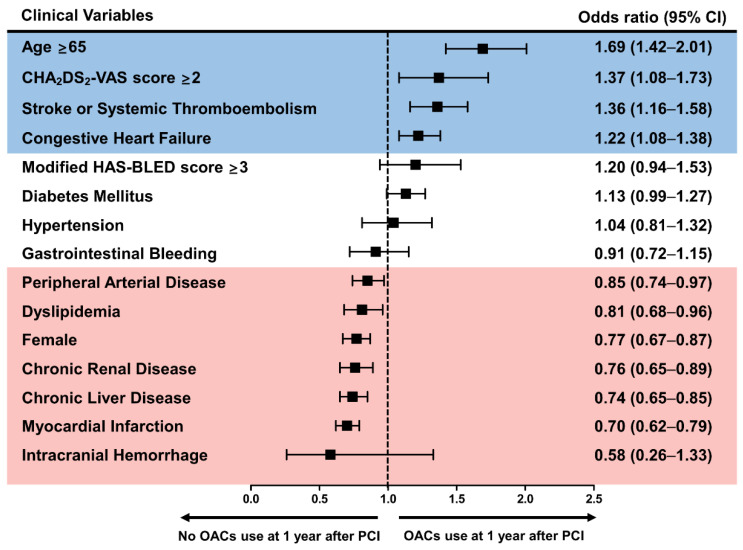
Clinical factors associated with preference for oral anticoagulants use 1 year after percutaneous coronary intervention. Old age (≥65 years), previous congestive heart failure, stroke or thromboembolism, and a high CHA_2_DS_2_-VASc score (≥2) were associated with OACs use at the 1-year period. In contrast, female sex, previous myocardial infarction, peripheral arterial disease, dyslipidemia, chronic liver or renal disease were associated with no OACs use at the 1-year period. CI: confidence interval; OACs: oral anticoagulants; PCI: percutaneous coronary intervention.

Old age (≥65 years), congestive heart failure, prior stroke or thromboembolism, and a high CHA_2_DS_2_-VASc score (≥2) were significantly associated with OACs use at the 1-year period (Figure 3 and Table S3). Among the predictors, old age showed the strongest association with OACs use (odds ratio (OR) 1.69, 95% confidence interval(CI) 1.42–2.01). In contrast, female sex, MI, PAD, dyslipidemia, and chronic liver or renal disease were significantly associated with no OACs use at the 1-year period, of which previous MI showed the strongest association (OR 0.70, 95% CI 0.62–0.79).

### 3.4. Clinical Factors Associated with a Preference for Oral Anticoagulant Monotherapy 1 Year after Percutaneous Coronary Intervention

**Figure 4:**
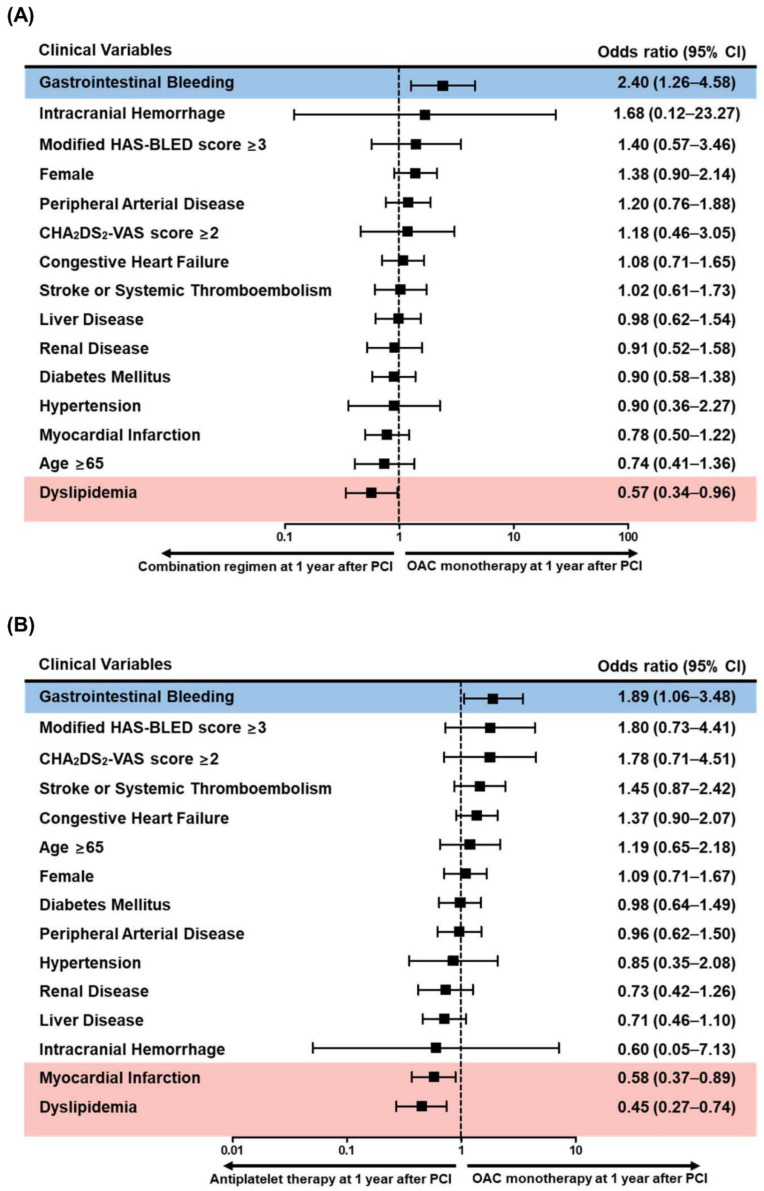
Clinical factors associated with preference for oral anticoagulant monotherapy compared with combination regimens or antiplatelet-only therapy 1 year after percutaneous coronary intervention. Among the clinical factors, previous gastrointestinal bleeding was associated with a preference for OAC monotherapy over combination regimens, whereas dyslipidemia was associated with a preference for combination regimens (**A**). Previous gastrointestinal bleeding was also associated a preference for OAC monotherapy than antiplatelet-only therapy. However, previous MI and dyslipidemia were associated with a preference for antiplatelet-only therapy (**B**). CI: confidence interval; OAC: oral anticoagulant; PCI: percutaneous coronary intervention.

With regard to specific antithrombotic therapy regimens, a history of GI bleeding was significantly associated with a preference for OAC monotherapy over combination regimens 1 year after PCI, and dyslipidemia was associated with a preference for combination regimens (Figure 4A and Table S4). A history of MI and dyslipidemia was associated with a preference for antiplatelet-only therapy over OAC monotherapy 1 year after PCI (Figure 4B and Table S5). Notably, a history of GI bleeding was associated with a preference for OAC monotherapy.

## 4. Discussion

NOACs are considered the mainstay of therapy for stroke prevention in patients with AF, and a growing body of evidence has reported beneficial outcomes with a NOAC-based combination regimen for patients with AF who undergo PCI [7,8,9,10]. Therefore, after PCI, guidelines recommend NOAC administration in preference to VKAs in patients who are eligible to receive NOACs as combination therapy that includes antiplatelet agents [1,2]. The current study investigated whether the updated guidelines have led to changes in the long-term treatment patterns of antithrombotic therapy among patients with AF who undergo PCI. We observed that the overall OAC prescription rate during the 2-year follow-up period after PCI significantly increased from the pre-NOACs to the NOAC era. However, the OAC prescription rate remained low after PCI in all three cohorts, and most patients received antiplatelet therapy without OACs. Our results highlight the significant gap between the current guidelines and real-world clinical practice, even after the transition to the NOAC era.

We observed that predictors of bleeding risk, including chronic liver and renal disease, were significantly associated with OAC non-use 1 year after PCI. Studies have reported that ethnicity may affect the bleeding risk associated with antithrombotic therapy; the risk is higher in Asian than in Western populations [11]. Based on our findings, we suggest that fear of bleeding events following combination therapy may discourage healthcare providers from prescribing OAC therapy over prolonged periods after PCI [12,13]. However, insufficient anticoagulation in patients with AF would further increase the thromboembolic risk [13]. Therefore, it is important to maintain a careful balance between the thromboembolic and bleeding risks, particularly in Asian patients with AF. A shorter duration of combination therapy based on the individual bleeding risks in addition to strategies to minimize PCI-induced bleeding (e.g., radial artery access) could be a useful approach for reducing bleeding complications [1,2].

Factors associated with a high risk of ischemic events often serve as predictors of major bleeding [14]. We observed that age and previous stroke were strongly associated with OAC use 1 year post-PCI. Moreover, these two factors have coincidentally been incorporated as components of the HAS-BLED score (an indicator of overall bleeding risk) [15]. Therefore, the positive trend observed with the HAS-BLED score for OAC therapy 1 year after PCI is attributable to the positive association between OAC therapy and age, as well as previous stroke. The increasing burden of risk factors for both thromboembolic and bleeding events is a major drawback that interferes with adherence to guideline-based antithrombotic therapy in clinical practice [14]. In view of the high rate of stent thrombosis or recurrent MI in the early phase after PCI, DAPT without OAC therapy or the use of novel antiplatelet agents over a short period could be considered to maximize the effectiveness of DAPT and to minimize the bleeding risk following the use of a combination regimen [16]. However, such strategies are not currently recommended by the guidelines, and to date, there is a lack of data to directly compare DAPT with a combination regimen in patients with AF who undergo PCI. Therefore, clinicians should follow evidence-based treatment guidelines and use an individualized approach based on patients’ thromboembolic and bleeding risks.

We observed that the baseline DAPT prescription rate was higher in patients with old MI or a history of peripheral artery disease. We have previously reported a negative association between vascular disease and triple antithrombotic therapy after PCI in patients with AF [4]. After PCI, those who received DAPT without OACs were significantly associated with OAC non-use 1 year after PCI [5]. Our current finding highlights trends observed in real-world clinical practice, which show a preference for maintenance of antiplatelet therapy without OACs in patients with a history of vascular disease. A significant burden of coronary disease or complex PCI procedures in patients with vascular disease could also affect the preference for antiplatelet therapy over a combination regimen after PCI [17]. However, studies have confirmed the beneficial outcomes of NOAC-based combination therapy, regardless of coronary lesion characteristics or procedural complexities [18].

Generally, triple therapy is recommended for 1–6 months after PCI, based on the patient’s risk of ischemia and bleeding [1,2]. However, bleeding risks that are known to be associated with triple therapy have led to the development of a new treatment regimen that comprises double antithrombotic therapy but excludes aspirin from the combination regimen [19]. After the introduction of NOACs, four randomized trials have reported that post-PCI NOAC-based double therapy is superior to VKA-based triple therapy with regard to bleeding events after PCI [7,8,9,10]. More recent evidence that supports double therapy has been adopted in the updated guidelines as a treatment option for patients with a high bleeding risk [1,2]. We observed a significant increase in the OAC prescription rates over 2-year post-PCI follow-up from the pre-NOAC to the NOAC era, which could be attributed to the increase in the percentage of NOAC prescriptions. High rates of post-PCI NOAC-based antithrombotic therapy have also been observed in other Asian populations [20,21]. In Korea, the reimbursement criteria for NOAC prescriptions in patients with AF were expanded in 2015, which could have contributed to the increase in the NOAC prescription rates in patients with AF who undergo PCI. However, 33.6% of patients who were administered OACs continued to receive triple therapy 1 year after PCI. This suggests a preference for 1-year post-PCI maintenance DAPT even after the widespread availability of NOACs, which resulted in overtreatment and noncompliance with the guideline recommendations [1,2]. Compared with double therapy, such overtreatment with prolonged triple therapy increases bleeding risk without additional benefit in reducing thromboembolic risk [22].

Following the administration of combination therapy using OACs and antiplatelet agents, life-long anticoagulation with OAC monotherapy for stroke prevention is recommended 1 year after PCI [1,2]. We observed a significantly low rate of OAC monotherapy 1 year after PCI in all three cohorts. Recently, the Atrial Fibrillation and Ischemic events with Rivaroxaban in patiEnts with stable coronary artery disease (AFIRE) study reported that rivaroxaban monotherapy was superior to combined therapy containing a single antiplatelet drug for bleeding risk and was non-inferior with regard to the risk of ischemia in patients with AF and stable coronary disease [23]. The Optimizing Antithrombotic Care in patients with AtriaL fibrillatiON and coronary stEnt (OAC-ALONE) trial that investigated patients with AF beyond 1 year post-PCI with coronary stenting compared the benefits of OAC monotherapy with a combination regimen [24]. However, the results were underpowered for the primary endpoint owing to early trial termination due to delayed enrollment. We observed that a history of MI and dyslipidemia was associated with a preference for antiplatelet-only therapy over OAC monotherapy at 1-year follow-up. However, OAC monotherapy was preferred over combination regimens in patients with a history of gastrointestinal (GI) bleeding. These results highlight a tendency observed in clinical practice to maintain antiplatelet therapy beyond 1 year after PCI for secondary prevention in those with MI but not in patients with bleeding events. AF in patients who undergo PCI is associated with advanced coronary disease [25]; therefore, healthcare providers are hesitant to discontinue antiplatelet therapy 1 year after PCI. Further studies are necessary to guide optimal antithrombotic therapy beyond 1 year after PCI in patients with AF.

Our results should be interpreted with caution owing to the following limitations of this study: (a) Owing to the non-randomized study design, we could not control actual patient compliance with prescribed medications across the study population. However, to our knowledge, the current study is the largest study that has investigated 2-year longitudinal patterns in antithrombotic therapy after PCI in patients with AF; this fact serves as a strength of this research. (b) The association between changes in OAC treatment patterns and long-term clinical outcomes were not investigated in this study. Future studies are necessary to investigate the clinical outcomes associated with different types of antithrombotic therapies in patients with AF who undergo PCI. (c) Indications for PCI, such as acute MI or stable angina, may affect the DAPT prescription rate. In this study, we selected patients with AF who underwent PCI from the claims database based on procedure codes. Therefore, we speculate that our study population would include patients with a variety of indications for PCI, including MI. (d) Up to 20% of patients did not receive any antithrombotic therapy at the 2-year follow-up period after PCI. A similar trend was reported by a previous nationwide study performed during the VKA era, which included 16.5% of patients with no antithrombotic therapy at the 2-year follow-up [5]. These results could be attributed to ethnic differences in the bleeding risk associated with antithrombotic therapy; the risk is higher among Asian than among Western patients [11]. We speculate that owing to the well-known risk of bleeding events associated with combination regimens, healthcare providers might be hesitant to continue prescribing OAC or antiplatelet therapy after PCI. Additionally, poor compliance with guideline-based antithrombotic treatment observed in clinical practice (which could be associated with a fear of bleeding complications) may at least partly contribute to the increase in the percentage of patients included in the post-PCI no-treatment group. However, data regarding major bleeding outcomes were unavailable in our study; therefore, we could not determine the exact percentage of patients included in the no-treatment group who truly developed bleeding complications. (e) The severity of coronary lesions and complexities of PCI procedures may affect baseline OAC prescription rates after PCI. However, information regarding the detailed coronary lesion characteristics and PCI procedures was unavailable in the HIRA database. Moreover, data regarding laboratory test results, such as serum creatinine levels are unavailable in the HIRA database; therefore, we could not confirm the label adherence to NOACs.

## 5. Conclusions

We observed low OAC prescription rates in Asian patients with AF who underwent PCI, even after the introduction of NOACs. Although the NOAC-based antithrombotic therapy prescription rates increased, most patients continued to receive antiplatelet therapy without OACs over the 2-year post-PCI follow-up. We observed a preference for prolonged DAPT, even in combination with OACs as triple therapy.

## Figures and Tables

**Table 1:** Baseline characteristics of the study population.

	(A) Pre-NOAC Era (*n* = 5044)	(B) Transition Era (*n* = 7209)	(C) NOAC Era (*n* = 6438)	A vs. B	A vs. C	B vs. C
**Demographics**						
Age, years	71 (63–77)	72 (63–78)	72 (63–78)	0.007	<0.001	0.434
Age groups				<0.001	<0.001	0.105
<65 years	1420 (28.2)	2056 (28.5)	1846 (28.7)			
65–74 years	1885 (37.4)	2444 (33.9)	2080 (32.3)			
≥75 years	1739 (34.5)	2709 (37.6)	2512 (39.0)			
Women	1775 (35.2)	2449 (34.0)	2167 (33.7)	0.487	0.259	0.999
**Comorbidities**						
Diabetes Mellitus	2041 (40.5)	2867 (39.8)	2516 (39.1)	0.999	0.398	0.999
Hypertension	4500 (89.2)	6503 (90.2)	5770 (89.6)	0.223	0.999	0.777
Dyslipidemia	4023 (79.8)	6173 (85.6)	5626 (87.4)	<0.001	<0.001	0.008
Congestive Heart Failure	1891 (37.5)	3064 (42.5)	3053 (47.4)	<0.001	<0.001	<0.001
Myocardial Infarction	1876 (37.2)	2895 (40.2)	2746 (42.7)	0.003	<0.001	0.009
Peripheral Arterial Disease	1360 (27.0)	1946 (27.0)	1824 (28.3)	0.999	0.312	0.243
Stroke/TIA/Systemic Thromboembolism	897 (17.8)	1196 (16.6)	1043 (16.2)	0.253	0.074	0.999
Intracranial Hemorrhage	43 (0.9)	52 (0.7)	39 (0.6)	0.999	0.358	0.999
Gastrointestinal Bleeding	443 (8.8)	580 (8.0)	458 (7.1)	0.440	0.003	0.121
Renal Disease	863 (17.1)	1407 (19.5)	1323 (20.5)	0.002	<0.001	0.397
Liver Disease	1816 (36.0)	2656 (36.8)	2600 (40.4)	0.999	<0.001	<0.001
CHA_2_DS_2_-VAS score	3 (2–5)	4 (2–5)	4 (2–5)	0.018	<0.001	0.012
0	83 (1.6)	108 (1.5)	84 (1.3)			
1	541 (10.7)	671 (9.3)	587 (9.1)			
2	946 (18.8)	1310 (18.2)	1121 (17.4)			
3	998 (19.8)	1465 (20.3)	1206 (18.7)			
4	874 (17.3)	1244 (17.3)	1141 (17.7)			
5 or higher	1602 (31.8)	2411 (33.4)	2299 (35.7)			
Modified HAS-BLED score	3 (3–4)	3 (3–4)	3 (3–4)	0.645	0.024	0.363
1	163 (3.2)	200 (2.8)	179 (2.8)			
2	827 (16.4)	1183 (16.4)	969 (15.1)			
3 or higher	4054 (80.4)	5826 (80.8)	5290 (82.2)			
**Baseline Antithrombotic Therapy after PCI**						
OACs overall	1256 (24.9)	1941 (26.9)	2263 (35.2)	0.036	<0.001	<0.001
VKAs	1235 (24.5)	1872 (26.0)	826 (12.8)	0.005	<0.001	<0.001
NOACs	21 (0.4)	69 (1.0)	1437 (22.3)	0.005	<0.001	<0.001
Triple therapy	1228 (24.3)	1886 (26.2)	2202 (34.2)	0.070	<0.001	<0.001
VKA-based	1207 (23.9)	1819 (25.2)	817 (12.7)	0.007	<0.001	<0.001
NOAC-based	21 (0.4)	67 (0.9)	1385 (21.5)	0.007	<0.001	<0.001
Double therapy (OACs + SAPT)	26 (0.5)	53 (0.7)	56 (0.9)	0.404	0.076	0.999
VKA-based	26 (0.5)	52 (0.7)	8 (0.1)	0.999	<0.001	<0.001
NOAC-based	0 (0.0)	1 (0.0)	48 (0.7)	0.999	<0.001	<0.001
DAPT	3697 (73.3)	5147 (71.4)	4093 (63.6)	0.063	<0.001	<0.001

Abbreviation: DAPT, dual antiplatelets; IQR, interquartile range; NOACs, non-vitamin K antagonist oral anticoagulants; OACs, oral anticoagulants; PCI, percutaneous coronary intervention; SAPT, single antiplatelet; SD, standard deviation; TIA, transient ischemic attack; VKAs, vitamin K antagonists. Values are given as median (interquartile range), or number (percentage), unless otherwise indicated.

## Data Availability

The original data underlying this article were derived from the Korean Health Insurance Review and Assessment Service (HIRA) claims database, which can be accessed via reasonable request through the Healthcare Bigdata Hub (https://opendata.hira.or.kr).

## Supplementary Materials

The following are available online at https://www.mdpi.com/article/10.3390/jcm10071505/s1, Tables: Table S1. Definition of comorbidity/scores/outcomes; Table S2. Two-year patterns in antithrombotic therapy among patients with atrial fibrillation after percutaneous coronary intervention; Table S3. Factors associated with oral anticoagulants use 1 year after percutaneous coronary intervention; Table S4. Factors associated with preference for oral anticoagulant monotherapy over combination regimens 1 year after percutaneous coronary intervention; Table S5. Factors associated with preference for oral anticoagulant monotherapy over antiplatelet only therapy 1 year after percutaneous coronary intervention. Figures: Figure S1. Sensitivity analysis of the 2-year patterns in antithrombotic therapy after percutaneous coronary intervention in patients with atrial fibrillation (excluding all censored cases from the study population). After excluding all censored cases, we found consistent trends for the 2-year patterns in antithrombotic therapy after PCI in pre-NOAC era (A), transition era (B), and NOAC era (C). DAPT: dual antiplatelet therapy; OAC: oral anticoagulant; N: non-vitamin K oral anticoagulant; PCI: percutaneous coronary intervention; SAPT: single antiplatelet therapy; W: warfarin.
