# Supplementary material for: Longitudinal Patterns in Antithrombotic Therapy in Patients with Atrial Fibrillation after Percutaneous Coronary Intervention in the Non-Vitamin K Oral Anticoagulant Era: A Nationwide Population-Based Study
Source: J Clin Med. 2021 Apr 4;10(7):1505. doi: 10.3390/jcm10071505 (PMC8038511; doi:10.3390/jcm10071505)
Supplement: Supplementary file 1 [file jcm-10-01505-s001.pdf]

SUPPLEMENTARY DATA

Table S1. Definition of comorbidity/scores/outcomes

Diagnosis	ICD-10-CM code and definition
**Inclusion criteria**	
Atrial fibrillation	I48.0-48.4, I48.9
Percutaneous coronary intervention	M6551-6552, M6561-6564, M6571-6572
**Comorbidities**	
Diabetes mellitus*	E11-E14; and minimum 1 prescription of anti-diabetic drugs (sulfonylureas, metformin, meglitinides, thiazolidinediones, dipeptidyl peptidase-4 inhibitors, α-glucosidase inhibitors and insulin).
Hypertension*	I10-I13, I15; and minimum 1 prescription of anti-hypertensive drug (thiazide, loop diuretics, aldosterone antagonist, alpha-/beta-blocker, calcium-channel blocker, angiotensin-converting enzyme inhibitor, angiotensin II receptor blocker).
Dyslipidemia	E78
Congestive heart failure	I50
Myocardial infarction	I21, I22
Peripheral artery disease	I70, I73
Stroke	I63, I64
Transient ischemic attack	G458, G459
Systemic thromboembolism	I26, I74, I802
Intracranial hemorrhage	I60-I62
Gastrointestinal bleeding	K22.6, K25.0, K25.2, K25.4, K25.6, K26.0, K26.2, K26.4, K26.6, K27.0, K27.2, K27.4, K27.6, K28.0, K28.2, K28.4, K28.6, K29.0, K62.5, K92.0, K92.1, K92.2
Renal disease	I13.1, N03, N05, N10-N19, Z49, Z94.0, Z99.2
Liver disease	K70, K72-K76, K71.3-K71.7
**Scores**	

CHA_2DS_2-VASc score	Heart failure (1 point), hypertension (1 point), age ≥75 years (2 points), diabetes (1 point), previous stroke/systemic embolism/transient ischemic attack (2 points), vascular disease (prior MI or PAD, 1 point) and female sex (1 point)
Modified HAS-BLED score†	Hypertension (1 point), liver disease (1 point), renal disease (1 point), stroke history (1 point), bleeding history (1 point), age>65 years (1 point) and drug (concomitant use of NSAID or antiplatelet agent, 1 point)

All variables except hypertension and diabetes mellitus are defined when patients had one or more diagnoses during hospitalization or at outpatient clinic.

* Hypertension and diabetes mellitus were identified when patients had ≥1 diagnosis during hospitalization or ≥2 diagnoses at outpatient clinic for preventing overestimation of diagnosis.

† Labile international normalized ratio (INR) and alcohol use could not be evaluated from claims and were excluded from scoring in this study.

Table S2. Two-year patterns in antithrombotic therapy among patients with atrial fibrillation after percutaneous coronary intervention

pre-NOACs era	3M-	6M	9M	12M	15M	18M	21M	24M	p-trend
**Total Number of Patients**	4,877	4,775	4,667	4,526	4,406	4,316	4,255	4,172	
**Censored Patients**	167	269	377	518	638	728	789	872	<0.001
Death	158	241	308	371	425	475	510	557	<0.001
repeated PCI	9	28	69	147	213	253	279	315	<0.001
Triple (VKAs-based)	1,236	701	541	440	346	203	152	130	<0.001
Triple (NOACs-based)	30	28	24	17	15	14	9	3	<0.001
Dual (VKAs-based)	20	184	245	295	385	470	481	436	<0.001
Dual (NOACs-based)	0	5	11	21	21	21	25	24	<0.001
VKAs monotherapy	2	42	50	57	59	92	116	132	<0.001
NOACs monotherapy	0	1	1	7	17	21	17	18	<0.001
Dual antiplatelets	3,553	3,401	3,222	2,994	2,560	2,005	1,774	1,468	<0.001
Single antiplatelet	30	212	266	323	586	1,031	1,189	1,114	<0.001
No treatment	6	201	307	372	417	459	492	847	<0.001
**Transition era**	**3M**	**6M**	**9M**	**12M**	**15M**	**18M**	**21M**	**24M**	**p-trend**
**Total Number of Patients**	6,933	6,788	6,618	6,424	6,242	6,107	5,974	5,877	
**Censored Patients**	276	421	591	785	967	1,102	1,235	1,332	<0.001
Death	251	368	476	565	639	725	808	866	<0.001
repeated PCI	25	53	115	220	328	377	427	466	<0.001
Triple (VKAs-based)	1,818	984	681	500	388	182	139	97	<0.001
Triple (NOACs-based)	126	123	132	146	172	117	119	73	0.732
Dual (VKAs-based)	37	308	431	512	551	613	549	489	0.001
Dual (NOACs-based)	2	56	124	189	280	405	473	447	<0.001
VKAs monotherapy	0	40	46	56	62	91	105	122	<0.001

NOACs monotherapy	0	13	25	42	76	111	159	198	<0.001
Dual antiplatelets	4,904	4,623	4,303	3,916	3,295	2,610	2,255	1,826	<0.001
Single antiplatelet	42	340	431	542	820	1,321	1,443	1,387	<0.001
No treatment	4	301	445	521	598	657	732	1,238	<0.001
**NOACs era**	**3M**	**6M**	**9M**	**12M**	**15M**	**18M**	**21M**	**24M**	**p-trend**
**Total Number of Patients**	6,184	6,008	5,847	5,662	5,494	5,385	5,298	5,226	
**Censored Patients**	254	430	591	776	944	1,053	1,140	1,212	<0.001
Death	240	363	448	528	601	657	711	760	<0.001
repeated PCI	14	67	143	248	343	396	429	452	<0.001
Triple (VKAs-based)	783	433	292	175	128	73	42	33	<0.001
Triple (NOACs-based)	1474	730	523	426	383	210	180	118	<0.001
Dual (VKAs-based)	7	154	230	273	267	283	288	250	0.002
Dual (NOACs-based)	42	458	646	762	832	926	913	794	0.001
VKAs monotherapy	2	14	19	26	28	39	41	56	<0.001
NOACs monotherapy	4	58	87	129	195	307	374	408	<0.001
Dual antiplatelets	3,842	3,654	3,317	2,987	2,470	1,934	1,705	1,386	<0.001
Single antiplatelet	25	244	354	445	706	1,096	1,183	1,076	<0.001
No treatment	5	263	379	439	485	517	572	1,105	<0.001

Abbreviation: M, months; NOACs, non-vitamin K oral anticoagulants; PCI, percutaneous coronary intervention; VKAs, vitamin-K antagonists.

Table S3. Factors associated with oral anticoagulants use 1 year after percutaneous coronary intervention

	No OACs (N=3,871)	OACs (N=1,791)	Univariate OR (95% CI)	Multivariate OR (95% CI)
**Age**				
<65	1,301 (33.6)	380 (21.2)	1(Ref.)	1(Ref.)
65≤	2,570 (66.4)	1,411 (78.8)	1.88 (1.60-2.21)	1.69 (1.42-2.01)
**Sex**				
Male	2,561 (66.2)	1,201 (67.1)	1(Ref.)	1(Ref.)
Female	1,310 (33.8)	590 (32.9)	0.96 (0.83-1.11)	0.77 (0.67-0.87)
**Diabetes Mellitus**				
No	2,452 (63.3)	1,085 (60.6)	1(Ref.)	1(Ref.)
Yes	1,419 (36.7)	706 (39.4)	1.12 (0.98-1.29)	1.13 (0.99-1.27)
**Hypertension**				
No	451 (11.7)	159 (8.9)	1(Ref.)	1(Ref.)
Yes	3,420 (88.3)	1,632 (91.1)	1.35 (1.07-1.71)	1.04 (0.81-1.32)
**Dyslipidemia**				
No	443 (11.4)	255 (14.2)	1(Ref.)	1(Ref.)
Yes	3,428 (88.6)	1,536 (85.8)	0.78 (0.64-0.95)	0.81 (0.68-0.96)
**Congestive Heart Failure**				
No	2,188 (56.5)	892 (49.8)	1(Ref.)	1(Ref.)
Yes	1,683 (43.5)	899 (50.2)	1.31 (1.14-1.50)	1.22 (1.08-1.38)
**Myocardial Infarction**				
No	2,182 (56.4)	1,159 (64.7)	1(Ref.)	1(Ref.)
Yes	1,689 (43.6)	632 (35.3)	0.70 (0.61-0.81)	0.70 (0.62-0.79)
**Peripheral Arterial Disease**				
No	2,794 (72.2)	1,316 (73.5)	1(Ref.)	1(Ref.)

Yes	1,077 (27.8)	475 (26.5)	0.94 (0.80-1.09)	0.85 (0.74-0.97)
**Stroke / TIA / Systemic Thromboembolism**				
No	3,327 (85.9)	1,453 (81.1)	1(Ref.)	1(Ref.)
Yes	544 (14.1)	338 (18.9)	1.42 (1.19-1.71)	1.36 (1.16-1.58)
**Intracranial Hemorrhage**				
No	3,847 (99.4)	1,783 (99.6)	1(Ref.)	1(Ref.)
Yes	24 (0.6)	8 (0.4)	0.72 (0.27-1.91)	0.58 (0.26-1.33)
**Gastrointestinal Bleeding**				
No	3,600 (93.0)	1,678 (93.7)	1(Ref.)	1(Ref.)
Yes	271 (7.0)	113 (6.3)	0.90 (0.68-1.18)	0.91 (0.72-1.15)
**Renal Disease**				
No	3,140 (81.1)	1,494 (83.4)	1(Ref.)	1(Ref.)
Yes	731 (18.9)	297 (16.6)	0.85 (0.71-1.02)	0.76 (0.65-0.89)
**Liver Disease**				
No	2,245 (58.0)	1,149 (64.2)	1(Ref.)	1(Ref.)
Yes	1,626 (42.0)	642 (35.8)	0.77 (0.67-0.89)	0.74 (0.65-0.85)
**CHA_2DS_2-VAS score**				
<2	479 (12.4)	152 (8.5)	1(Ref.)	1(Ref.)
2≤	3.392 (87.6)	1,639 (91.5)	1.52 (1.21-1.92)	1.37 (1.08-1.73)
**Modified HAS-BLED score**				
<3	808 (20.9)	256 (14.3)	1(Ref.)	1(Ref.)
3≤	3,063 (79.1)	1,535 (85.7)	1.58 (1.31-1.91)	1.20 (0.94-1.53)

Hosmer and Lemeshow Goodness-of-Fit test: $\chi^2 = 12.8$, p = 0.119

Abbreviation: CI, confidence interval; OACs, oral anticoagulants; OR, odds ratio; TIA, transient ischemic attack.

Table S4. Factors associated with preference for oral anticoagulants monotherapy over combination regimens 1 year after percutaneous coronary intervention

	Combination regimens (N=1,636)	OACs monotherapy (N=155)	Univariate OR (95% CI)	Multivariate OR (95% CI)
**Age**				
<65	346 (21.1)	34 (21.9)	1(Ref.)	1(Ref.)
65≤	1,290 (78.9)	121 (78.1)	0.96 (0.59-1.55)	0.74 (0.41-1.36)
**Sex**				
Male	1,108 (67.7)	93 (60.0)	1(Ref.)	1(Ref.)
Female	538 (32.3)	62 (40.0)	1.40 (0.93-2.11)	1.38 (0.90-2.14)
**Diabetes Mellitus**				
No	989 (60.5)	96 (61.9)	1(Ref.)	1(Ref.)
Yes	647 (39.5)	59 (38.1)	0.94 (0.62-1.42)	0.90 (0.58-1.38)
**Hypertension**				
No	147 (9.0)	12 (7.7)	1(Ref.)	1(Ref.)
Yes	1,489 (91.0)	143 (92.3)	1.18 (0.56-2.48)	0.90 (0.36-2.27)
**Dyslipidemia**				
No	222 (13.6)	33 (21.3)	1(Ref.)	1(Ref.)
Yes	1,414 (86.4)	122 (78.7)	0.58 (0.35-0.96)	0.57 (0.34-0.96)
**Congestive Heart Failure**				
No	820 (50.1)	72 (46.5)	1(Ref.)	1(Ref.)
Yes	816 (49.9)	83 (53.5)	1.16 (0.78-1.73)	1.08 (0.71-1.65)
**Myocardial Infarction**				
No	1,051 (64.2)	108 (69.7)	1(Ref.)	1(Ref.)
Yes	585 (35.8)	47 (30.3)	0.78 (0.51-1.21)	0.78 (0.50-1.22)
**Peripheral Arterial Disease**				

No	1,208 (73.8)	108 (69.7)	1(Ref.)	1(Ref.)
Yes	428 (26.2)	47 (30.3)	1.23 (0.79-1.90)	1.20 (0.76-1.88)
**Stroke / TIA / Systemic Thromboembolism**				
No	1,329 (81.2)	124 (80.0)	1(Ref.)	1(Ref.)
Yes	307 (18.8)	31 (20.0)	1.08 (0.66-1.79)	1.02 (0.61-1.73)
**Intracranial Hemorrhage**				
No	1,629 (99.6)	154 (99.4)	1(Ref.)	1(Ref.)
Yes	7 (0.4)	1 (0.6)	1.51 (0.12-19.54)	1.68 (0.12-23.27)
**Gastrointestinal Bleeding**				
No	1,543 (94.3)	135 (87.1)	1(Ref.)	1(Ref.)
Yes	93 (5.7)	20 (12.9)	2.46 (1.32-4.60)	2.40 (1.26-4.58)
**Renal Disease**				
No	1,365 (83.4)	129 (83.2)	1(Ref.)	1(Ref.)
Yes	271 (16.6)	26 (16.8)	1.02 (0.59-1.74)	0.91 (0.52-1.58)
**Liver Disease**				
No	1,052 (64.3)	97 (62.6)	1(Ref.)	1(Ref.)
Yes	584 (35.7)	58 (37.4)	1.08 (0.71-1.63)	0.98 (0.62-1.54)
**CHA_2DS_2-VAS score**				
<2	142 (8.7)	10 (6.5)	1(Ref.)	1(Ref.)
2≤	1,494 (91.3)	145 (93.5)	1.38 (0.61-3.09)	1.18 (0.46-3.05)
**Modified HAS-BLED score**				
<3	238 (14.5)	18 (11.6)	1(Ref.)	1(Ref.)
3≤	1,398 (85.5)	137 (88.4)	1.30 (0.70-2.41)	1.40 (0.57-3.46)

Hosmer and Lemeshow Goodness-of-Fit test: $\chi^2 = 8.3$, $p = 0.403$

Abbreviation: CI, confidence interval; OACs, oral anticoagulants; OR, odds ratio; TIA, transient ischemic attack.

Table S5. Factors associated with preference for oral anticoagulants monotherapy over antiplatelet only therapy 1 year after percutaneous coronary intervention

	Antiplatelets only (N=3,432)	OACs monotherapy (N=155)	Univariate OR (95% CI)	Multivariate OR (95% CI)
**Age**				
<65	1,209 (35.2)	34 (21.9)	1(Ref.)	1(Ref.)
65≤	2,223 (64.8)	121 (78.1)	1.94 (1.21-3.10)	1.19 (0.65-2.18)
**Sex**				
Male	2,310 (67.3)	93 (60.0)	1(Ref.)	1(Ref.)
Female	1,122 (32.7)	62 (40.0)	1.37 (0.92-2.05)	1.09 (0.71-1.67)
**Diabetes Mellitus**				
No	2,181 (63.5)	96 (61.9)	1(Ref.)	1(Ref.)
Yes	1,251 (36.5)	59 (38.1)	1.07 (0.72-1.61)	0.98 (0.64-1.49)
**Hypertension**				
No	404 (11.8)	12 (7.7)	1(Ref.)	1(Ref.)
Yes	3,028 (88.2)	143 (92.3)	1.59 (0.77-3.29)	0.85 (0.35-2.08)
**Dyslipidemia**				
No	373 (10.9)	33 (21.3)	1(Ref.)	1(Ref.)
Yes	3,059 (89.1)	122 (78.7)	0.45 (0.28-0.73)	0.45 (0.27-0.74)
**Congestive Heart Failure**				
No	1,979 (57.7)	72 (46.5)	1(Ref.)	1(Ref.)
Yes	1,453 (42.3)	83 (53.5)	1.57 (1.06-2.33)	1.37 (0.90-2.07)
**Myocardial Infarction**				
No	1,952 (56.9)	108 (69.7)	1(Ref.)	1(Ref.)
Yes	1,480 (43.1)	47 (30.3)	0.57 (0.38-0.88)	0.58 (0.37-0.89)
**Peripheral Arterial Disease**				

No	2,476 (72.1)	108 (69.7)	1(Ref.)	1(Ref.)
Yes	956 (27.9)	47 (30.3)	1.13 (0.74-1.73)	0.96 (0.62-1.50)
**Stroke / TIA / Systemic Thromboembolism**				
No	2,990 (87.1)	124 (80.0)	1(Ref.)	1(Ref.)
Yes	442 (12.9)	31 (20.0)	1.69 (1.03-2.77)	1.45 (0.87-2.42)
**Intracranial Hemorrhage**				
No	3,409 (99.3)	154 (99.4)	1(Ref.)	1(Ref.)
Yes	23 (0.7)	1 (0.6)	0.96 (0.08-11.11)	0.60 (0.05-7.13)
**Gastrointestinal Bleeding**				
No	3,193 (93.0)	135 (87.1)	1(Ref.)	1(Ref.)
Yes	239 (7.0)	20 (12.9)	1.98 (1.09-3.59)	1.89 (1.06-3.48)
**Renal Disease**				
No	2,821 (82.2)	129 (83.2)	1(Ref.)	1(Ref.)
Yes	611 (17.8)	26 (16.8)	0.93 (0.55-1.57)	0.73 (0.42-1.26)
**Liver Disease**				
No	1,986 (57.9)	97 (62.6)	1(Ref.)	1(Ref.)
Yes	1,446 (42.1)	58 (37.4)	0.82 (0.55-1.23)	0.71 (0.46-1.10)
**CHA_2DS_2-VAS score**				
<2	451 (13.1)	10 (6.5)	1(Ref.)	1(Ref.)
2≤	2,981 (86.9)	145 (93.5)	2.19 (1.00-4.83)	1.78 (0.71-4.51)
**Modified HAS-BLED score**				
<3	759 (22.1)	18 (11.6)	1(Ref.)	1(Ref.)
3≤	2,673 (77.9)	137 (88.4)	2.16 (1.18-3.96)	1.80 (0.73-4.41)

Hosmer and Lemeshow Goodness-of-Fit test: $\chi^2 = 6.5$, $p = 0.596$

Abbreviation: CI, confidence interval; OACs, oral anticoagulants; OR, odds ratio; TIA, transient ischemic attack.

Figure S1

(A)

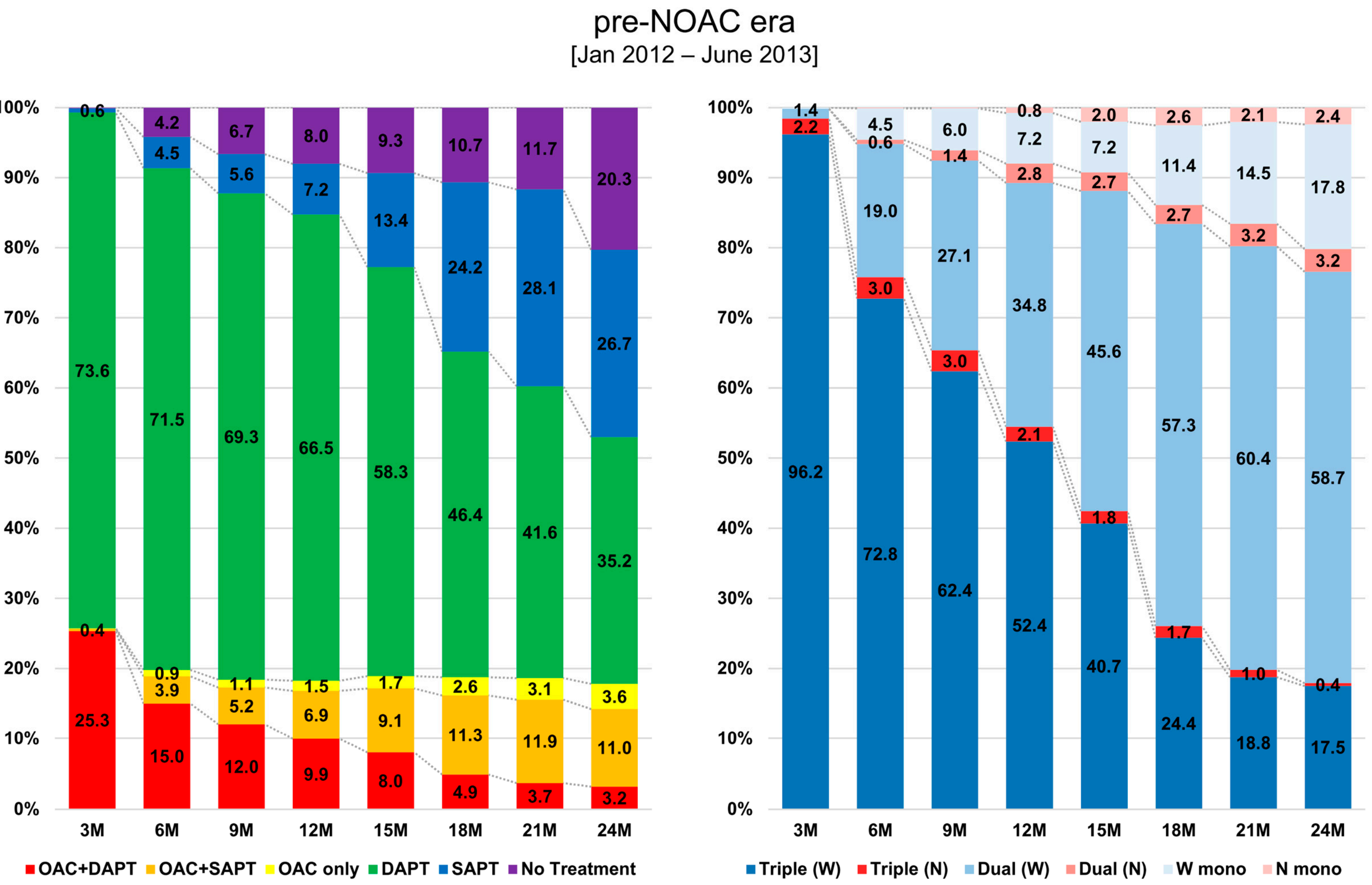
pre-NOAC era
[Jan 2012 – June 2013]
100%
90%
80%
70%
60%
50%
40%
30%
20%
10%
0%
3M
6M
9M
12M
15M
18M
21M
24M
OAC+DAPT
OAC+SAPT
OAC only
DAPT
SAPT
No Treatment
Triple (W)
Triple (N)
Dual (W)
Dual (N)
W mono
N mono

(B)

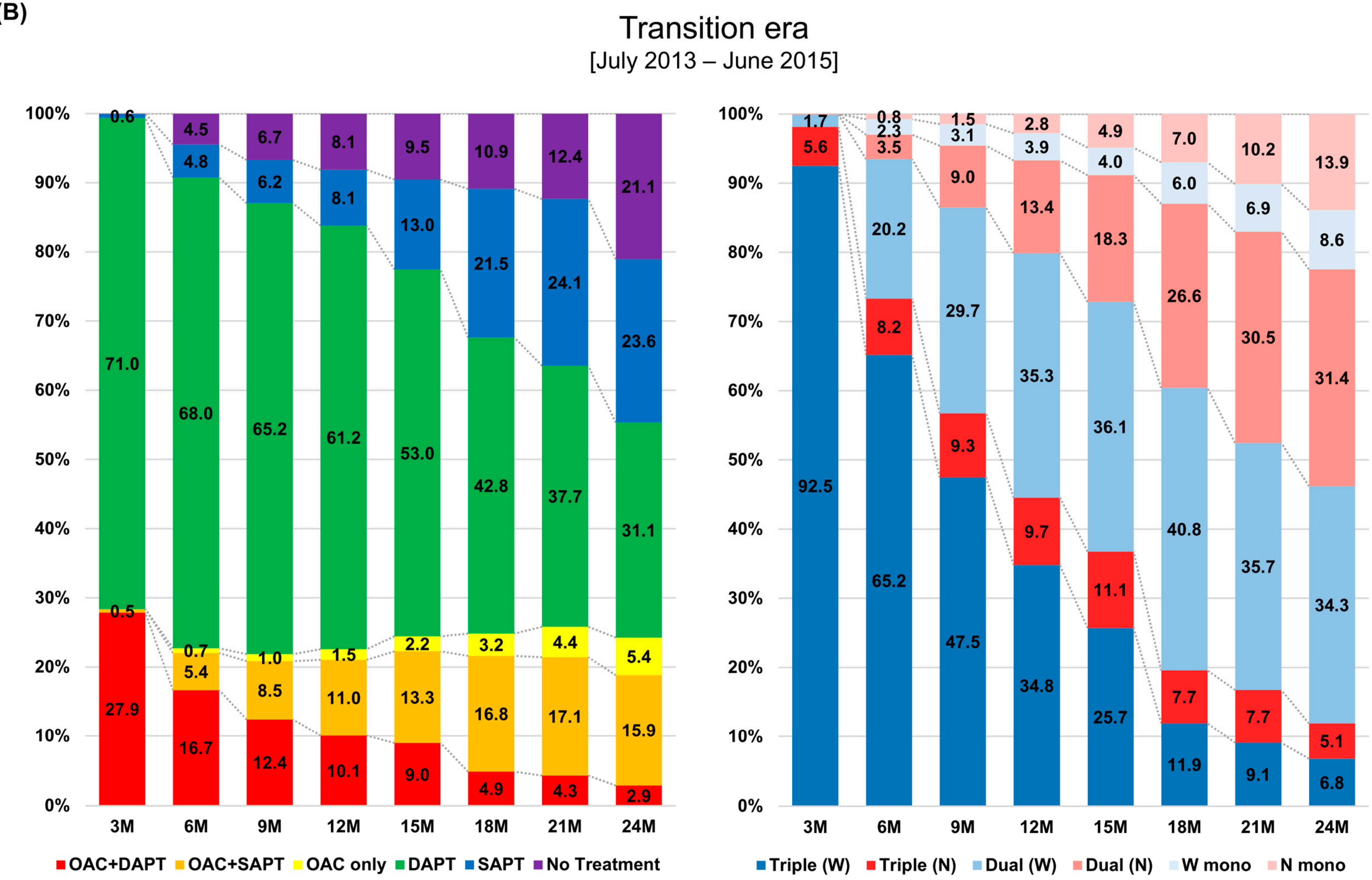
Transition era
[July 2013 – June 2015]
100%
90%
80%
70%
60%
50%
40%
30%
20%
10%
0%
3M
6M
9M
12M
15M
18M
21M
24M
0.6
71.0
0.5
27.9
4.5
4.8
68.0
0.7
5.4
16.7
6.7
6.2
65.2
1.0
8.5
12.4
8.1
8.1
61.2
1.5
11.0
10.1
9.5
13.0
53.0
2.2
13.3
9.0
10.9
21.5
42.8
3.2
16.8
4.9
12.4
24.1
37.7
4.4
17.1
4.3
21.1
23.6
31.1
5.4
15.9
2.9
OAC+DAPT
OAC+SAPT
OAC only
DAPT
SAPT
No Treatment
1.7
5.6
92.5
0.8
2.3
3.5
20.2
8.2
65.2
1.5
3.1
9.0
29.7
9.3
47.5
2.8
3.9
13.4
35.3
9.7
34.8
4.9
4.0
18.3
36.1
11.1
25.7
7.0
6.0
26.6
40.8
7.7
11.9
10.2
6.9
30.5
35.7
7.7
9.1
13.9
8.6
31.4
34.3
5.1
6.8
Triple (W)
Triple (N)
Dual (W)
Dual (N)
W mono
N mono

(C)

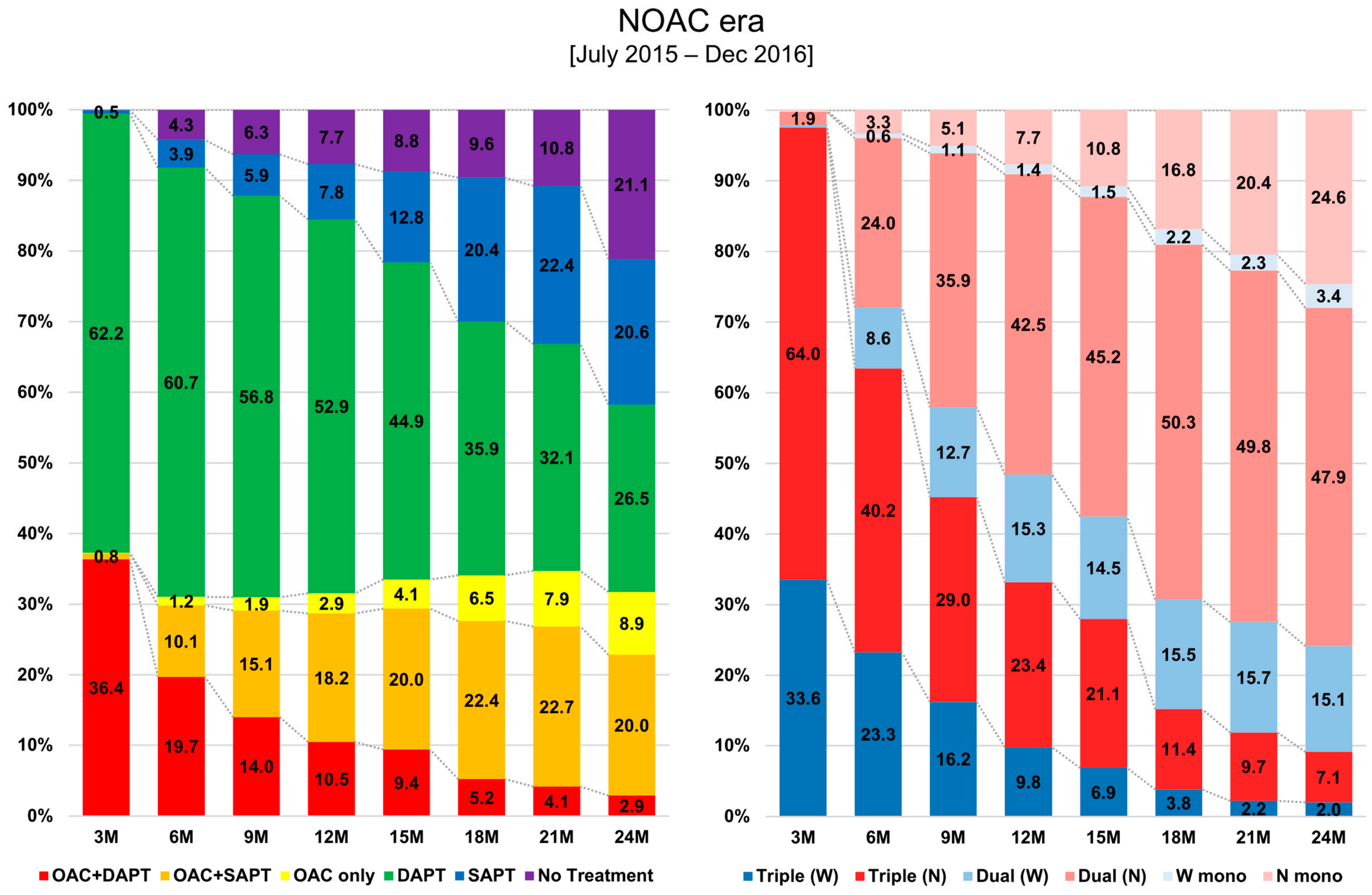
NOAC era
[July 2015 – Dec 2016]
100%
90%
80%
70%
60%
50%
40%
30%
20%
10%
0%
3M
6M
9M
12M
15M
18M
21M
24M
OAC+DAPT
OAC+SAPT
OAC only
DAPT
SAPT
No Treatment
Triple (W)
Triple (N)
Dual (W)
Dual (N)
W mono
N mono
